# A diabetes prediction model based on Boruta feature selection and ensemble learning

**DOI:** 10.1186/s12859-023-05300-5

**Published:** 2023-06-01

**Authors:** Hongfang Zhou, Yinbo Xin, Suli Li

**Affiliations:** 1 School of Computer Science and Engineering, Xi’an University of Technology, Xi’an, 710048 China; 2 Shaanxi Key Laboratory of Network Computing and Security Technology, Xi’an, 710048 China

**Keywords:** Diabetes detection, Machine learning, Boruta feature selection, K-Means++, Ensemble learning

## Abstract

**Background and objective:**

As a common chronic disease, diabetes is called the “second killer” among modern diseases. Currently, there is no medical cure for diabetes. We can only rely on medication for auxiliary treatment. However, many diabetic patients still die each year. In addition, a considerable number of people do not pay attention to their physical health or opt out of treatment due to lack of money, which eventually leads to various complications. Therefore, diagnosing diabetes at an early stage and intervening early is necessary; thus, developing an early detection method for diabetes is essential.

**Methods:**

In this study, a diabetes prediction model based on Boruta feature selection and ensemble learning is proposed. The model contains the use of Boruta feature selection, the extraction of salient features from datasets, the use of the K-Means++ algorithm for unsupervised clustering of data and stacking of an ensemble learning method for classification. It has been validated on a diabetes dataset.

**Results:**

The experiments were performed on the PIMA Indian diabetes dataset. The model was evaluated by accuracy, precision and F1 index. The obtained results show that the accuracy rate of the model reaches 98% and achieves good results.

**Conclusion:**

Compared with other diabetes prediction models, this model achieved better results, and the obtained results indicate that this model is superior to other models in diabetes prediction and has better performance.

## Introduction

**Fig. 1:**
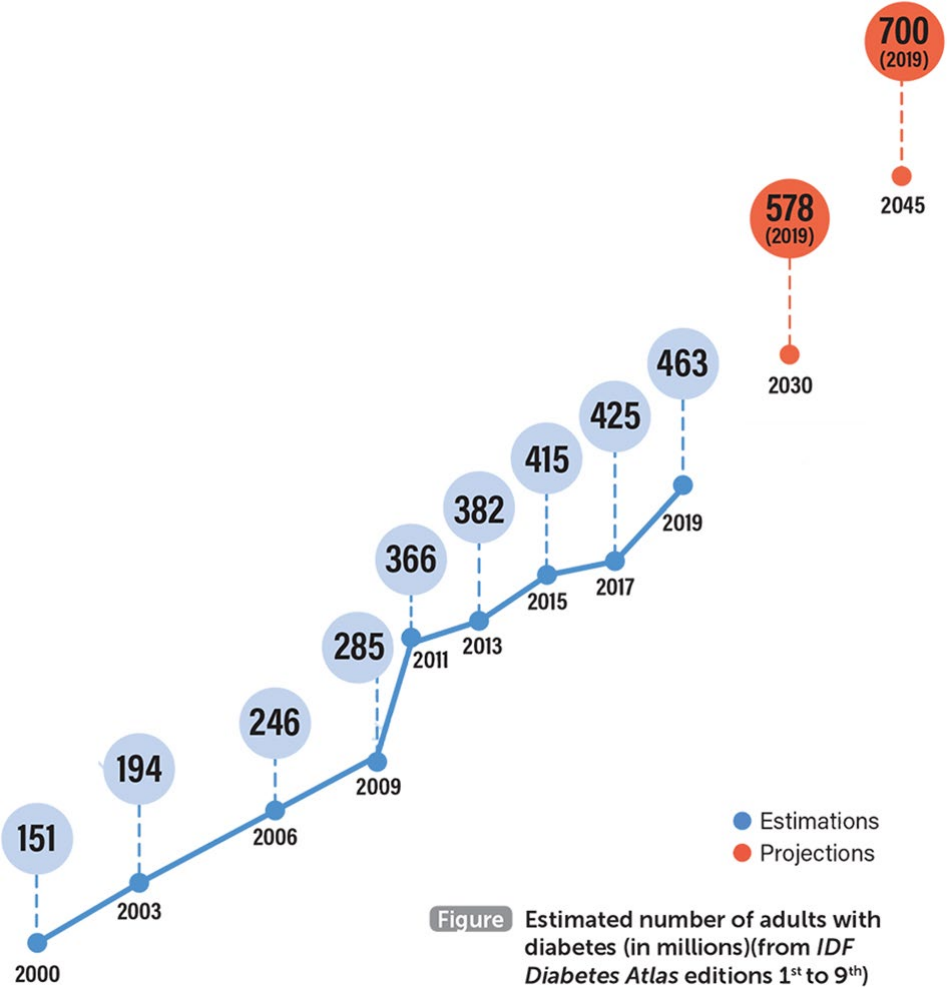
Global diabetes growth curve

With the rapid development of the social economy, people’s quality of life has constantly improved, and the diet structure has also significantly changed. Therefore, a variety of chronic diseases arise, and diabetes is one of the most common. Insulin is a hormone that regulates blood glucose homeostasis. When the pancreas does not produce enough insulin or the body does not use the produced insulin effectively, blood sugar rises, leading to hyperglycemia, which can lead to diabetes. With time, this can cause serious damage to the human body and result in blindness, amputation, heart disease, stroke, and kidney failure. Diabetes incidence is second only to cancer, and it is known as the “second killer” among modern diseases [1]. In the 2019 Global Leading Cause of Death Survey, diabetes was included in the top 10 causes of death [2]. According to the International Diabetes Federation, 700 million adults in the world will have diabetes by 2045. The cost of healthcare for diabetes is significant, at approximately $760 billion annually. The global growth curve of the number of people with diabetes is shown in Fig. 1 below (Image source: Statistics from the International Diabetes Federation) [3].

With the expansion of artificial intelligence applications, especially in disease diagnosis and medical image processing, it has become possible to use machine learning techniques to extract valid information from medical data for predicting chronic diseases. If we can predict the diabetic population and nondiabetic population at an early stage, when doctors diagnose diabetes, they can tend to focus on people with a high probability of having diabetes, which greatly reduces the intervention of human factors and provides a general direction for doctors to diagnose and take timely measures related to prevention and interception. It will be of great benefit to reduce the incidence of diabetes, improve people’s quality of life and the healthy life expectancy of the population, and will also effectively reduce the burden of diabetes treatment. This is the most fundamental motivation for us to carry out this work.

Ensemble learning is mainly a combination of several single classifiers in different ways and is used to improve the accuracy and robustness of classification. There are three main types: bagging, boosting and stacking. The bagging method subsamples from the training set to form the required subtraining set for each base model and combines the results predicted by all base models to produce the final prediction results. The boosting method is trained in the order of the base models, and if the previous base model has incorrect classification results, then the next base model can be trained with a larger weight assigned to correct the classification results, and the results predicted by all base models are linearly combined to produce the final prediction results. The stacking method is mainly divided into a base model and a meta-model. By training the base model, the generated results are used as input to the meta-model, which is trained to produce the final classification results.

Research on combinatorial classifiers has been conducted in areas such as disease prediction and bioinformatics. Leyi Wei et al. [4] proposed a PIHS algorithm based on selective integration learning, which combines the prediction results of each basic model by voting and uses a partitioning strategy to achieve a high level of performance on several biological informatics problems, showing high efficiency and robustness. Cheng Chen et al. [5] proposed a prediction framework called StackPPI, using XGBboost to reduce feature noise. An ensemble classifier using a combination of random forest, random tree and logistic regression algorithms is used as a classifier, and the method mainly works on protein and drug design with good classification performance. Jasmina Nali´c et al. [6] proposed a hybrid data mining model based on a combination of multiple feature selection and ensemble learning classification algorithms, which used a soft voting approach to synthesize classifiers into eight different ensemble models. Finally, GLM + DT’s model had the best hybrid performance, which was later tested on biological datasets and outperformed other ensemble learning models and single classifiers. Rajesh Yakkundimath et al. [7] proposed a new classifier combination model for the classification of cervical cancer cells, which uses Artificial Neural Network(ANN), Random Forest (RF) and Support Vector Machine (SVM) as basic classifiers. It is more suitable for the classification of cervical cancer cells compared to the results achieved by a single basic classifier. Tien Thanh Nguyen et al. [8] proposed a combinatorial classifier based on a Bayesian inference framework, which estimates a multivariate Gaussian distribution for each class of data using a variational inference approach, which was tested on 18 datasets and a medical imaging database and compared with several well-known ensemble methods, resulting in a large advantage.

Combined classifiers perform better than single classifiers. When performing classification, we always want to find a classification model with high robustness, high accuracy and balanced time complexity and space complexity. However, this is a relatively ideal state. When we perform classification, the classifier will be more or less influenced by the dataset, such as extremes, outliers and other noisy data, which can affect the classification results. Once noisy data are present, the performance of the single classifier will be greatly degraded. In contrast, with a combination classifier, different weights will be assigned according to the votes, and the misclassified data can be reclassified, and the combination classifier also has good adaptability to noisy data; thus, a combinatorial classifier was adopted in this study. Because diabetes is a common chronic disease, the classification of diabetic patients differs from other datasets. Alternatively, the classification of medical datasets differs from the classification of other datasets because the diagnosis of a particular disease is a rigorous, long-term process that involves people economically, physically and psychologically, especially for chronic diseases. If a disease is misdiagnosed, it can be fatal for the patient. Therefore, for the diagnosis of diseases, the choice of classifier has higher requirements, as aspects such as accuracy are very important. For the research direction proposed in this paper, for the classification of the diabetic population, experiments on single and combined classifiers have shown that the combined classifier has better results.

In this paper, a diabetes prediction model based on Boruta feature selection and ensemble learning is proposed. The model uses the Boruta feature selection algorithm, K-Means++ unsupervised cluster learning algorithm and stacking ensemble learning method.

There are three main contributions in this paper.

1. Feature Selection. In this thesis, the main focus is on the prediction of diabetes. For the diabetes dataset, it is necessary to determine the attributes that best match the diagnosis of diabetes, and we consider the attributes selected by a comprehensive comparison with Boruta’s algorithm through the selection methods for different features, such as Pearson’s correlation coefficient and PCA, as the most appropriate.
2. A clustering algorithm was used on the data. To provide the correct number of clusters, we used the K-Means +  + algorithm, which is an improved version of K-Means. The K-Means +  + algorithm optimises some of the problems that exist in the K-Means algorithm [27].
3. The most suitable base classifier and meta-classifier were selected, and the ensemble learning stacking method was used and tested repeatedly to determine the most suitable parameter values for diabetes. Most of them use single models for classification, such as Support Vector Machine (SVM), Logistic Regression(LR) or Softmax, but single models are highly susceptible to noisy data if they are not sufficiently trained, resulting in poor prediction accuracy. Additionally, most of the research workers did not adjust the parameters of the model. Thus, are the parameters at this time in line with the optimal parameters for diabetes prediction? Based on this, we first selected the most suitable base classifier and original classifier for the stacking method of ensemble learning. Second, the values of the focal parameters of the metamodel and the base model were determined by repeated experiments.

The subsequent organization of this paper is as follows. An overview of the work conducted by other researchers in diabetes prediction is presented in Section II. The model proposed in this paper and the methods used in the model are described in Section III, including Boruta feature selection, K-Means++, and ensemble learning. The experimental procedure is described in detail in Section IV, including dataset description, steps, parameter settings, etc. Section V discusses the experimental results, including evaluation of the model, comparison with other models, and comparative experiments. Section VI summarizes the work and provides some suggestions for future work.

## Literature survey

In this section, we review the work done by other researchers in diabetes prediction using machine learning and deep learning methods.

### Machine learning

Chen et al. [9] analyzed the relationship between diabetes and the levels of several elements in hair/urine samples for the diagnosis of diabetes, and principal component analysis was used to perform preliminary processing work on the data. Both ensemble learning and Support Vector Machine (SVM) algorithms were used as classifiers with an average accuracy, sensitivity and specificity of 99%, 100%, and 99% and 97%, 89%, and 99% for hair and urine samples, respectively. Finally, it was shown by various model evaluation metrics that hair samples are superior to urine samples for the diagnosis and prevention of diabetes and that they provide more valuable information for the prevention, diagnosis, treatment and research of diabetes.

Perveen et al. [10] proposed an AdaBoost and bagging integration technique based on J48 (c4.5) using J48 (c4.5) as the base classifier and combining the standalone data mining technique J48 to classify diabetic patients. Tested on the Canadian primary care surveillance network dataset, the experimental results show that the AdaBoost integration method outperforms bagging and independent J48 decision trees. In addition, the researchers propose that Naive Bayesian (NB), Support Vector Machine (SVM), etc., can be used as the basic learning algorithms in the ensemble learning framework, and the method can be applied to other disease datasets such as hypertension, coronary heart disease, etc.

Wu, Yang et al. [11] proposed a new model for predicting type 2 diabetes mellitus (T2DM) based on data mining techniques, which consists of a modified K-Means algorithm and a logistic regression algorithm. The improved K-Means algorithm addresses the randomness of the seed values by inserting a procedure to record and sort the values called the “sum of squared errors within clusters” in ascending order; the smaller is the value, the better is the result. The model was evaluated on the PIMA Indian diabetes dataset as well as on two other diabetes datasets with good experimental results, and the prediction accuracy of the model was 3.04% higher than that of other researchers.

Zhu et al. [12] proposed an improved logistic regression model for diabetes prediction by integrating PCA and K-Means techniques, which provided adequate and efficient clustered datasets. The model consists of three components: principal component analysis, K-Means and logistic regression algorithms, and data normalization. The experimental results showed that PCA enhanced the accuracy of the K-Means clustering algorithm and logistic regression classifier compared to other published findings, with K-Means outputting 25 correctly classified data points and logistic regression accuracy improving by 1.98%.

Lukmanto et al. [13] used F-exponential feature selection and fuzzy Support Vector Machine for the detection and classification of diabetes. Feature selection was used to extract valuable features from the dataset. Then, the dataset was trained using SVM, fuzzy rules were generated, and finally, the output was classified using the fuzzy inference method. The method achieves an accuracy of 89.02% on the PIMA Indian Diabetes dataset. Moreover, the employed method provides an optimized fuzzy rule count while still maintaining sufficient accuracy.

Shankar G et al. [14] proposed a diabetes prediction model based on fuzzy logic with the gray wolf optimizer algorithm. The fuzzy rules are learned by the model and then optimized according to the GWO algorithm and validated on the dataset with an accuracy of 81%. The proposed model is based on the gray wolf optimization algorithm, which is able to globally optimize the features and gives higher accuracy than the ant colony algorithm.

Beschi Raja et al. [15] proposed a predictive model for type 2 diabetes based on a data mining strategy consisting of particle swarm optimization (PSO) and fuzzy clustering (FCM). It was evaluated by conducting experiments on the PIMA Indian diabetes dataset and using sensitivity, specificity and accuracy metrics. The obtained results showed that the accuracy of the model was improved by 8.26% compared to other methods, and the model had better performance compared to other methods.

Howsalya Devi et al. [16] proposed a diabetes diagnosis method combining a furthest-first (FF) clustering algorithm and a sequence minimum optimization (SMO) classifier algorithm. The clustering algorithm divides the data into different sets of clusters at first, which reduces the size of the dataset and greatly shortens the computation time. Then, the clustering output is used as the input of SVM to complete the classification. The method achieved better results on the PIMA Indian Diabetes dataset. The experimental results show that the ensemble method has 99.4% accuracy in predicting diabetes. The experimental results prove that the hybrid approach of data mining methods can help doctors make better clinical diagnosis decisions for diabetic patients.

Saloni et al. [17] proposed binary classification using an ensemble soft voting classifier and completed the classification using the ensemble of three machine learning algorithms (random forest, logistic regression and Naive Bayes). In this paper, the proposed method is experimentally evaluated using the proposed method and basic classifiers (AdaBoost, Logistic, SVM, RF, Naive Bayes, Bagging, GradientBoost, XGBoost, CatBoost). The accuracy, precision, recall, and F1 index were used as evaluation criteria. The values of accuracy, precision, recall, and F1 index for the PIMA Indian diabetes dataset were 79.04%, 73.48%, 71.45%, and 80.6%, respectively.

Jobeda Jamal Khanam et al. [18]used seven machine learning and neural network algorithms to predict diabetes on the PIMA diabetes dataset. And neural network models with different hidden layers for different periods were built.The experimental results showed that the models using Logistic Regression (LR) and Support Vector Machine (SVM) were beneficial for diabetes prediction, and the accuracy of neural networks with two hidden layers is 88.6%.

Rajendra et al. [19] compared logistic regression algorithms and ensemble learning techniques for diabetes prediction and conducted experiments on the PIMA diabetes dataset.The experimental results show that logistic regression is one of the effective algorithms for building predictive models.This study also found that the use of data pre-processing, feature selection and integration techniques could also improve the accuracy of the model.

Rawat et al. [20] conducted comparative experiments on the PIMA diabetes dataset based on machine learning algorithms such as Naïve Bayesian (NB), Support Vector Machine (SVM), and Neural Network. The experimental results showed that the neural network was the best classifier with an accuracy of 98%.Therefore, the neural network approach is the best way to detect diabetic disease at an early stage.

Su et al. [21] used XGBoost, LightGBM, Neural Network, Logistic Regression algorithms for joint data modeling between different organizations. They conducted on PIMA diabetes dataset. The experimental results show that using federated learning models we can make better use of the patient data between different organizations and deliver a reliable and improved prediction of Diabetes Mellitus risks.

### Deep learning

Edla et al. [22] proposed a deep neural network framework using stacked autoencoders for the classification of diabetes data, using stacked autoencoders to extract features from the dataset and using softmax to complete the classification. The method uses accuracy, recall and F1 index as evaluation metrics. The accuracy and recall for the PIMA Indian diabetes dataset were 90.66% and 87.92%, respectively.

Nguyen et al. [23] applied a broad deep learning model that combines the strengths of generalized linear models with various features and deep feedforward neural networks to improve prediction of the onset of type 2 diabetes mellitus (T2DM). Our final ensemble model not using SMOTE obtained an accuracy of 84.28%, area under the receiver operating characteristic curve (AUC) of 84.13%, sensitivity of 31.17% and specificity of 96.85%, further optimizing the prediction of diabetes onset.

Rahman et al. [24] proposed a novel diabetes classification model based on convolutional long short-term memory (CONV-LSTM). The method was tested on the PIMA Indian diabetes dataset and compared with three models: convolutional neural network (CNN), traditional LSTM (T-LSTM) and CNN-LSTM, and the obtained results showed an accuracy of up to 97.26%, outperforming the other three models and the state-of-the-art model.

Bala et al. [25] developed a deep neural network (DNN) classifier, an unsupervised learning approach, which is used for accurate prediction for the Pima Indian diabetes dataset, and a feature importance model that is bagged with extra trees and random forest is used for feature selection. The model achieved 98.16% accuracy with a random train-test split, and it was observed that the model obtained better performance than other state-of-art methods.

Garc´ıa-Ord´as et al. [26] proposed a method based on deep learning techniques for predicting diabetic patients. The method includes data enhancement using a variational autoencoder (VAE), feature enhancement using a sparse autoencoder (SAE) and a convolutional neural network for classification. Feature extraction was performed on the PIMA Indian diabetes dataset, considering information such as the number of pregnancies, glucose levels, insulin levels, blood pressure, and age of the patients. The obtained results showed that the method achieved an accuracy of 92.31%, which was 3.17% more accurate than other methods.

Satish et al. [27] proposed a related technique for feature selection. The method applies AdaBoost to selected features for classification, and a novel stacking technique based on multilayer perceptron, Support Vector machine and logistic regression (MLP, SVM and LR) is designed and developed for the selected features. Its proposed stacking technique integrates intelligent models, improves model performance, and overcomes the decision residual problem that occurs with AdaBoost. The obtained results outperform other reported techniques based on the PIMA Indian diabetes dataset implementation.

Aghila et al. [28] proposed a custom hybrid model of an artificial neural network (ANN) and genetic algorithm for an efficient prediction framework of diabetic diseases. The method correctly identifies the importance of the impact of each variable on the output, thus prioritizing the variables considered to be the most important. The model and its corresponding decision algorithm achieved a prediction accuracy of 80% on the PIMA India diabetes dataset.

YalinWu et al. [29] proposed a new and efficient binary logistic regression (BLR) to accurately predict the specific type of T2DM and make the model adaptive to multiple datasets. To improve the recognition rate of the database, a series of preprocessing steps was performed, including outlier removal, normalization and missing value processing. The generated high-dimensional features were modeled using a BLR application. Experiments were conducted using XGBoost-BLR on the PIMA Indian diabetes dataset and early diabetes dataset with diabetes prediction identification rates of 94% and 98%, respectively.

Roobini et al. [30]used the Convolutional Graph Long Short Term Memory (CGLSTM) classifier for classification. The weights of this deep neural network were optimised using the AdaGrad optimiser to improve the accuracy of the predictions. They conducted experiments on the PIMA diabetes dataset and compared them with existing methods to demonstrate the efficiency of the proposed system.

Rabhi et al. [31] developed a generic deep-learning-based framework for modeling IMTS. This framework facilitated the comparative studies of sequential neural networks (transformers and long short-term memory) and irregular time representation techniques. This study highlighted the significance of modeling time gaps between medical records to improve prediction performance and the utility of a generic framework for conducting extensive comparative studies.

Qi et al. [32] proposed an ensemble learning framework: KFPredict, which combines multi input models with key features and machine learning algorithms. They first propose a multi-input neural network model (KF_NN) that fuses key features. Then, they ensemble KF_NN with three machine learning algorithms (i.e., Support Vector Machine, Random Forest and K-Nearest Neighbors) for soft voting to form our predictive classifier for diabetes prediction. Taking the PIMA diabetes dataset as the test data, the experiment shows that the framework presents good prediction results.

## Proposed methodology

This thesis proposes a diabetes prediction model based on Boruta feature selection and ensemble learning based on correlation work.

**Fig. 2:**
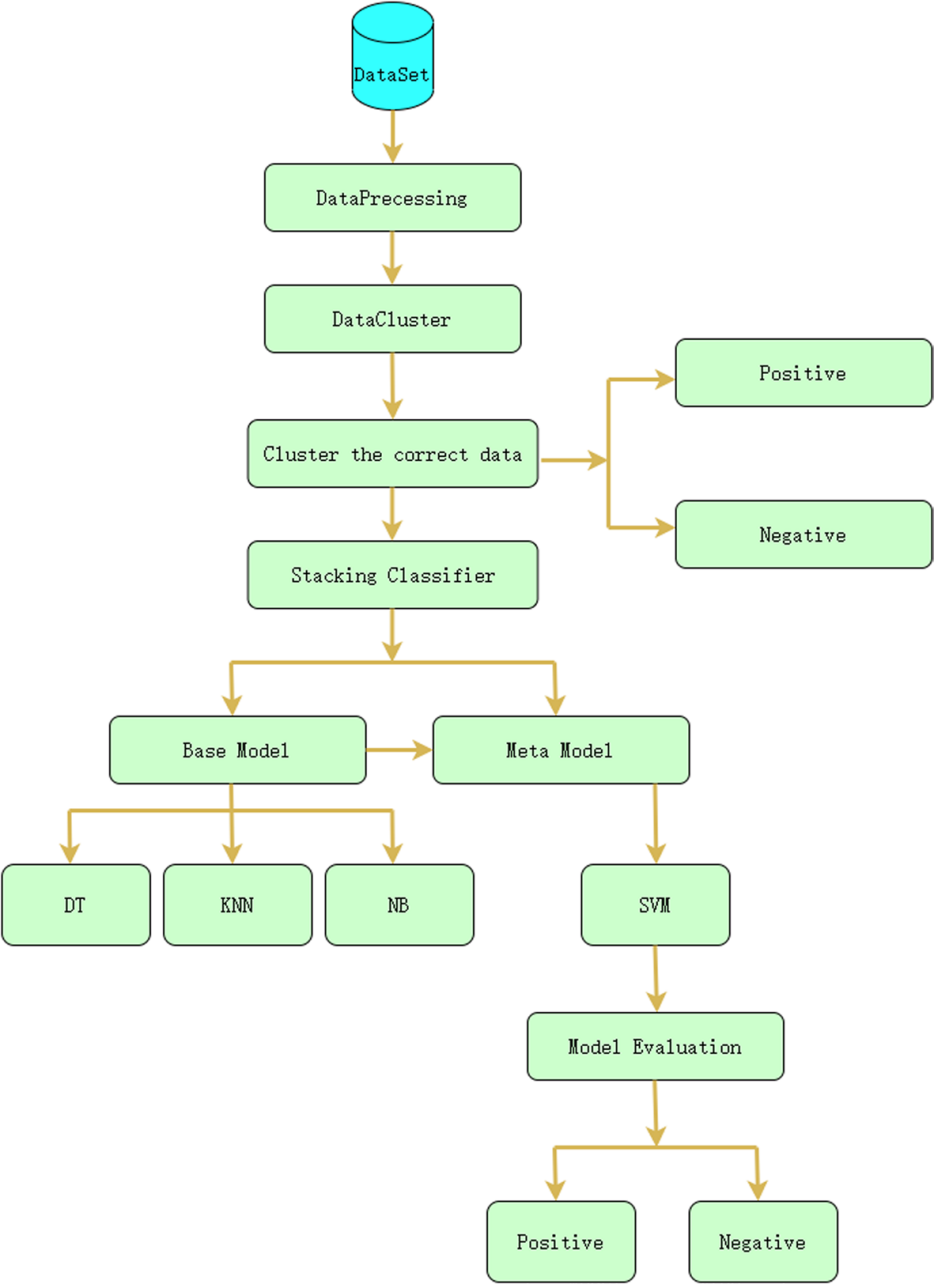
Model structure diagram

The model mainly uses the Boruta feature selection algorithm to select the features in the dataset, selecting the most relevant features for diabetes diagnosis and eliminating irrelevant features. In the unlabeled dataset, there are potentially K patterns in general; thus, we used the K-Means++ algorithm for unsupervised cluster learning on the dataset and found that the K patterns present in the dataset can be clustered into different clusters. Finally, data classification is performed using the stacking method in ensemble learning. Stacking in this paper uses Naive Bayesian (NB), K-Nearest Neighbor (KNN) and Decision Tree (DT) as the base model and Support Vector Machine (SVM) as the meta model. The specific steps of the model are shown in Fig. 2 below. After the original dataset is input, the dataset is preprocessed, and the results of the preprocessing are put through a clustering algorithm to calculate the correctly clustered data. The correctly clustered data are input into the stacking algorithm for classification. The results of the base model classification are fed into the meta-model, yielding diabetic and nondiabetic patients. The algorithms used in the model proposed in this paper are described below.

### Boruta feature selection

Boruta is a feature selection algorithm based on a random forest classifier. Unlike the goal of a general feature selection algorithm, the goal of the Boruta feature selection algorithm is to select the set of features that are most relevant to the dependent variable rather than to a particular model. Unlike the goal of a general feature selection algorithm, the goal of the Boruta feature selection algorithm is to select the set of features that are most relevant to the dependent variable rather than to select the minimum compact set of features for which a particular model is best suited. The specific steps of the Boruta feature selection algorithm are as follows [33].

1. Create a new feature matrix. Each feature of the real feature matrix M is randomly disordered to obtain the shadow feature matrix M_S. Then, we splice the shaded feature matrix M_S with the original feature matrix M to form a new feature matrix N, N = [M,M_S].
2. Use the feature matrix N as input and train the model and output the Feature_Importances model.
3. Calculate the Z_Score metric for the true feature matrix M and the shadow feature matrix M_S. Find the Z_Score metric with the largest shadow feature, denoted as ${\text{Z}}_{{{\text{max}}}}$.
4. Real features with Z_Score greater than ${\text{Z}}_{\max }$ are marked as”important” and real features with Z_Score less than ${\text{Z}}_{\max }$ are marked as insignificant” and removed from the feature set.
5. Remove all shadow features.
6. Repeat steps 1-5 until importance has been assigned to all features or the algorithm has reached the previously set number of random forest runs.

In this study, using the PIMA Indian diabetes dataset, the Boruta feature selection algorithm was used to select five features with high predictive relevance from eight features associated with diabetes prediction, namely, glucose, BMI, age, diabetes spectrum function, and insulin.

### K-means++ 

There are generally K potential patterns in the dataset. K-Means is a classical unsupervised cluster learning algorithm that finds K patterns in a dataset and uses the Euclidean distance as a measure of similarity. Generally, the closer is the distance, the greater is the similarity, and the farther is the distance, the lower is the similarity. However, the convergence of the K-Means algorithm is heavily dependent on the initialization status of the cluster centers. If all (or most) cluster centers are unfortunately initialized to the same cluster during the initialization process, then the K-Means clustering algorithm will largely fail to converge to the global optimal solution in this case. To solve this problem, the K-Means++ algorithm improves K-Means: when initializing the K cluster centers, the more distant are the samples from other cluster centers, the more likely are they to be selected as the next cluster center, thus solving the defective problem in the K-Means algorithm [34].

For better cluster learning, in this study, we use a modified version of the K-Means++ algorithm for unsupervised clustering learning. The specific implementation steps are shown below.

1. Create K points as the initial center-of-mass points (select the K data points with the greatest distance).
2. For each data point, the distance between it and the center-of-mass point is calculated, and the data point is assigned to the cluster with the closest distance, as shown in Eq. 1.
  1 $${\mathrm{D}}^{(\mathrm{i},\mathrm{j})}={\mathrm{argmin}}_{\mathrm{j}}{||{\mathrm{X}}^{\left(\mathrm{i}\right)}-{\upmu }_{(\mathrm{j})}||}^{2}$$
  where ${\mathrm{X}}^{\left(\mathrm{i}\right)}$ is the ith sample data point, ${\upmu }_{(\mathrm{j})}$ is the jth centroid, and ${\mathrm{D}}^{(\mathrm{i},\mathrm{j})}$ is the minimum distance between the sample data point and the centroid.
3. Determine whether the clusters where the sample points are located before and after clustering are the same; if they are, the algorithm terminates. Otherwise, go to step 4.
4. Calculate their respective centroids (Eq. 2) based on the sample points in each cluster, use the result of the calculation as the new centroid for that cluster, and go to step 2. The algorithm ends when the sample points in each cluster are not changing, i.e., when the convergence state is reached is the jth centroid. The centroid count function counts the number of sample points that belong to the current centroid.
  2 $${{\upmu }^{{\prime}}}_{\left(\mathrm{j}\right)}=\frac{{\sum }_{\mathrm{i}=1}^{\mathrm{m}}({\mathrm{X}}^{(\mathrm{i})}\in {\upmu }_{(\mathrm{j})})}{\mathrm{count}\left[{\sum }_{\mathrm{i}=1}^{\mathrm{m}}({\mathrm{X}}^{(\mathrm{i})}\in {\upmu }_{(\mathrm{j})})\right]}$$
  where ${{\upmu }^{^\prime}}_{\left(\mathrm{j}\right)}$ is the jth cluster’s new center point, ${\mathrm{X}}^{(\mathrm{i})}$ is the ith sample data point, and ${\upmu }_{(\mathrm{j})}$In this study, unsupervised cluster learning is performed using the K-Means++ algorithm by preprocessing the dataset with operations such as removing extremes and outliers, filling in missing values, and normalizing the data. By comparison with the original dataset, the correctly clustered data account for approximately 74% of the total data. These diabetic data will be used as the input for the ensemble learning stacking method.

### Ensemble learning

Stacking is an ensemble learning method that combines multiple classification models with a single meta-classifier. Stacking first obtains several base models based on different algorithms by parallel training, then combines the output of each base model by training a metamodel, and finally takes the output of the metamodel as the final output. Stacking in this paper uses NB, KNN and DT as the base model and SVM as the metamodel. The code of the stacking method is shown in Algorithm 1 below.
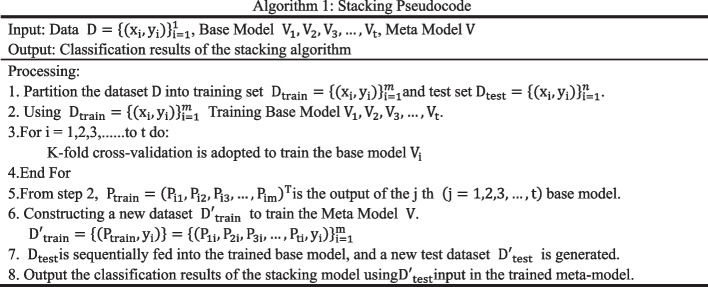


Where $\mathrm{D}$ is the dataset, ${\mathrm{x}}_{\mathrm{i}}$ is each sample data, and ${\mathrm{y}}_{\mathrm{i}}$ is the label corresponding to each sample data, ${\mathrm{D{^\prime}}}_{\mathrm{test}}=\left\{\left({\mathrm{P}}_{\mathrm{test}},{\mathrm{y}}_{\mathrm{i}}\right)\right\}={\left\{\left({\mathrm{P}}_{1\mathrm{i}},{\mathrm{P}}_{2\mathrm{i}},{\mathrm{P}}_{3\mathrm{i}},\dots ,{\mathrm{P}}_{\mathrm{ni}},{\mathrm{y}}_{\mathrm{i}}\right)\right\}}_{\mathrm{i}=1}^{\mathrm{n}}$, ${\mathrm{P}}_{\mathrm{test}}={\left({\mathrm{P}}_{\mathrm{i}1},{\mathrm{P}}_{\mathrm{i}2},{\mathrm{P}}_{\mathrm{i}3},\dots {,\mathrm{P}}_{\mathrm{in}}\right)}^{\mathrm{T}}$ is the output of the jth $\left(\mathrm{j}=\mathrm{1,2},3,\dots ,\mathrm{t}\right)$ base model.

### Grid search

In machine learning algorithms, the difference in parameters directly determines the effectiveness of a model. If the manual trial parameter approach is adopted, it is true that the optimal parameters can be obtained after a finite number of steps, but it will be labor-intensive and inefficient. To improve efficiency, reduce human error and be able to find the optimal parameters in the fastest way, grid search is used to select the optimal parameters in this study. The grid search method is an exhaustive search method for specifying parameter values. The method tries the possibility of each parameter by iterating through each parameter in a loop over the range of all parameter candidates and tests the model on the validation set. Finally, the parameter with the best model effect is the result of the final grid search and is the optimal parameter for the model within the range of parameter candidates. The grid search method ensures that the best model parameters are found within the candidate range of parameters.

Because the grid search method is an exhaustive approach that requires traversal of all possible parameter combinations, it can be time-consuming for large datasets and models with multiple parameters. The PIMA Indian diabetes dataset used in this study is a small dataset, and the model has relatively few parameters; thus, it is appropriate to use the grid search method to find the optimal parameters of the model.

## Experiment

### Dataset

This experiment used the PIMA Indian diabetes dataset, a common dataset for diabetes prediction.

#### Dataset description

The experiments used the PIMA Indian diabetes dataset from the UCI Machine Learning Repository, a common dataset for diabetes prediction. The dataset consisted of 768 women with and without diabetes from Arizona, USA, who were all over 21 years of age and had type 2 diabetes. The dataset includes nine attributes, eight of which are related to diabetes diagnosis (pregnancy, body mass index, insulin levels, age, blood pressure, skin thickness, glucose and diabetes spectrum function) and one label attribute. The label attribute is used to distinguish between diabetic and nondiabetic populations. The dataset consisted of 268 test-positive examples and 500 test-negative examples. The attribute values are specifically described as shown in Table 1 below.

**Table 1:** Dataset description

NO	Property name	Property description	Type of data	Data range	Missing value
1	Pregnancy	Number of female pregnancies	Integer	0–17	No
2	BMI	BMI (kg/m^2^)	Float	0–67.1	Yes
3	Insulin	2-h serum insulin	Integer	0–846	Yes
4	Age	Year	Integer	21–81	No
5	Blood pressure	Diastolic blood pressure (mmHg)	Integer	0–122	Yes
6	Skin thickness	Triceps skinfold thickness (mm)	Integer	0–99	Yes
7	Glucose	2-h blood glucose (mg/dl)	Integer	0–199	Yes
8	Diabetes spectrum function	Diabetes spectrum function	Float	0.078–2.42	No
9	Outcome	Diabetic population marker	Integer	0.1	No

The missing value, maximum value, minimum value, mean value, and standard deviation of each attribute in the dataset were counted, and the statistical results are shown in Table 2.

**Table 2:** Dataset statistics

NO	Attributes	Number of missing values	Average value	Maximum	Minimum	Standard deviation
1	Pregnancy	0	3.845	17	0	3.37
2	BMI	11	32.457	67.1	18.2	6.925
3	Insulin	374	155.548	846	14	118.776
4	Age	0	33.241	81	21	11.76
5	Blood pressure	35	72.405	122	24	12.382
6	Skin thickness	227	29.153	99	7	10.477
7	Glucose	5	121.687	199	44	30.536
8	Diabetes spectrum	0	0.472	2.42	0.078	0.331

### Data preprocessing

This experiment uses the WEKA data analysis tool to preprocess the data. WEKA is known as Waika to Intelligent Analysis Environment, an open source machine learning and data mining software based on the JAVA environment [35].

#### Handling missing data

From the description of the dataset, it can be seen that there are five attributes with missing values in the dataset, namely, body mass index, insulin level, blood pressure, skin thickness and glucose. The average value can better reflect the overall situation of a set of data; thus, the average value of the five attributes is taken to replace the missing values separately.

#### Handling noisy data

In this experiment, outliers and extreme values were processed by quartile analysis. By analyzing the outliers and extreme values in the dataset, 71 data points, including 45 outlier data points and 26 extreme value data points, were removed from the dataset, and 699 data samples were retained.

#### Boruta feature selection

In this experiment, we used the Boruta feature selection algorithm to select five features from the PIMA Indian diabetes dataset, namely, glucose, insulin level, body mass index, diabetes spectrum function and age. The data corresponding to the five features were saved and further processed.

#### Data standardization

In this experiment, we use the Z-Score method to standardize the data. Z-Score standardization is a data standardization based on the mean and standard deviation of the original data, and the standardized data are normally distributed, i.e., the distribution with mean 0 and standard deviation 1. The formula for Z-Score standardization is shown in Eq. 3.

3 $${\mathrm{x}}^{*}=\frac{\mathrm{x}-\upmu }{\upsigma }$$

where ${\mathrm{x}}^{*}$ represents the standardized data, $\mathrm{x}$ is the original data, $\upmu$ represents the average value of data, and $\sigma$ represents the standard deviation of data.

### Experimental procedure

We preprocess the data, select the features of the dataset using the Boruta feature selection algorithm, and use the normalized processed data as input for the subsequent processing. This experiment uses Python as the programming language, which has good portability, extensibility and interpretability. The computer parameters used in the experiments are as follows: CPU @1.90 GHZ, memory 16 GB, SSD 100 GB, etc.

#### K-means++ algorithm

For certain K potential patterns present in the dataset, we use the K-Means++ algorithm to cluster the data into K different clusters. Only two types of populations exist in the PIMA Indian diabetes dataset used in this experiment, namely, the diabetic and nondiabetic populations; thus, K is 2, i.e., divided into two different clusters. The unsupervised clustering learning of the data was performed using the K-Means++ algorithm and compared with the original dataset data, and finally, a total of 514 data were correctly clustered, including 332 nondiabetic population sample data and 182 diabetic population sample data. The proportion of correctly clustered data was calculated using the following formula. The unsupervised clustering learning of the data was performed using the K-Means++ algorithm and compared with the original dataset data, and finally, a total of 514 data were correctly clustered, including 332 nondiabetic population sample data and 182 diabetic population sample data. Equation 4 is used to calculate the proportion of correct data for clustering.

4 $$\mathrm{p}=\frac{\upvarepsilon }{\mathrm{n}}$$

where $\mathrm{p}$ is the proportion of correct data, $\upvarepsilon$ represents the number of correctly clustered sample data, and $\mathrm{n}$ represents the total number of sample data.

As seen above, the number of correctly clustered sample data accounts for approximately 74% of the total number of samples, and the 514 correctly clustered data are used as the input for stacking learning.

#### Ensemble learning stacking methods

We use stacking to perform ensemble learning classification on data that are correctly clustered. In this experiment, we use NB, KNN and DT as base models and SVM as a metamodel. The 514 data correctly clustered by the K-Means++ algorithm were fed into the stacking method for classification prediction, and the classification results were evaluated. The model parameters will be discussed in the next section.

### Parameter settings

In this experiment, we use grid search to select the parameters, specify the selection range of each parameter, evaluate the performance of the model by randomly combining various parameters by enumeration, and finally output the parameters when the model performance is optimal, and the corresponding parameters are also the optimal parameters at this time. For the three methods in stacking, NB, KNN and DT all use grid search to find the optimal parameters, and the specific parameters are debugged as shown in the figure below.

#### KNN parameter

In this experiment, the optimization of KNN parameters mainly includes the selection of K values (the number of proximity points) and Weights.

**Fig. 3:**
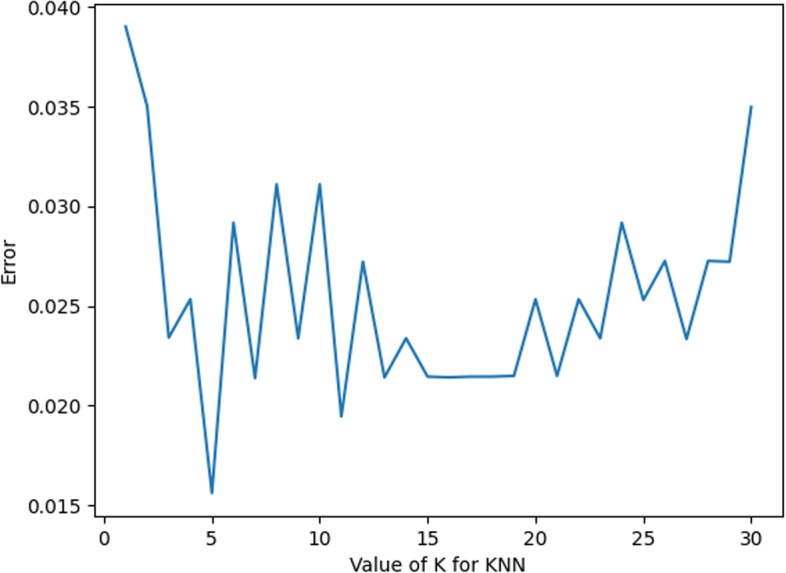
K value error plot

For the K value, as shown in Fig. 3 below, it can be found that when the K value is 5, the error value at this time is the smallest; thus, for the choice of K value, it is more appropriate to choose 5 in this experiment.

**Fig. 4:**
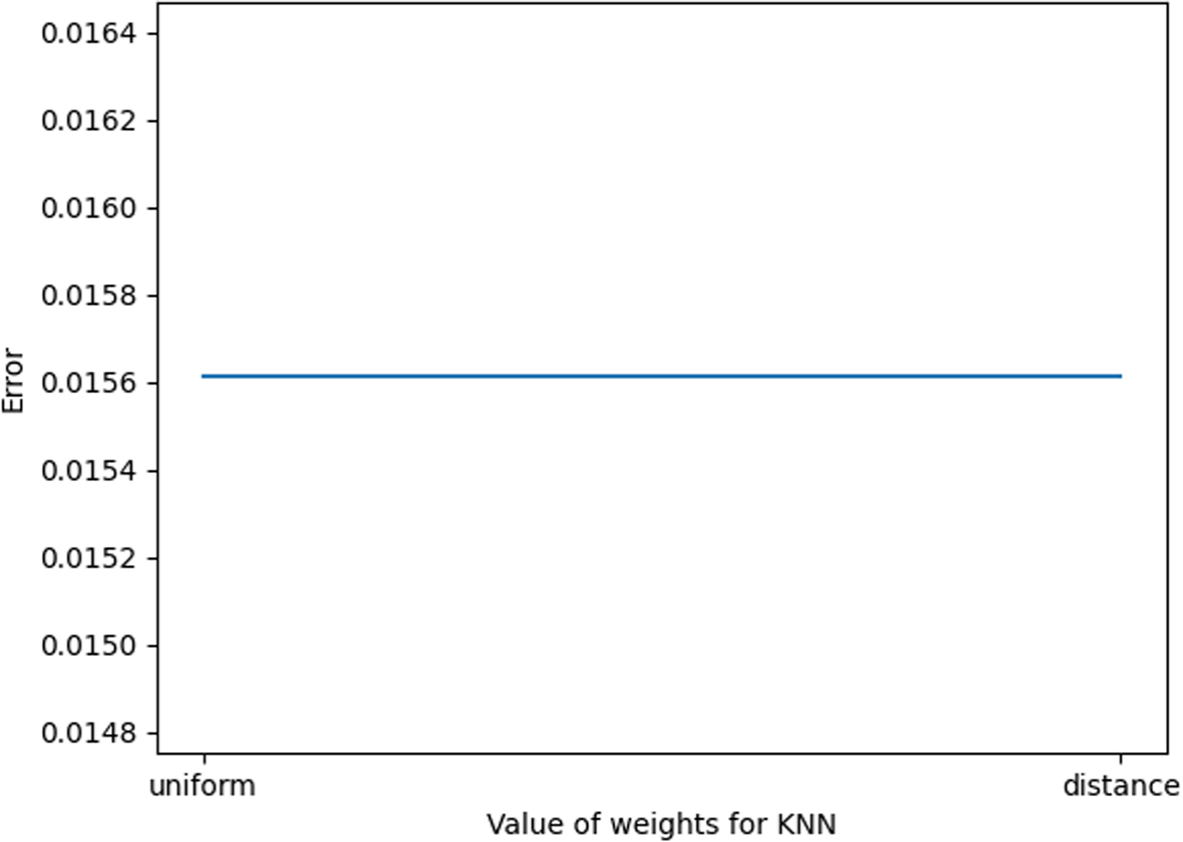
Weights value error plot

For Weights, as shown in Fig. 4 below, when Weights are selected as”uniform” and”distance”, the error values are the same. Taking this into account, “uniform” is chosen as the value of Weights in this experiment.

**Fig. 5:**
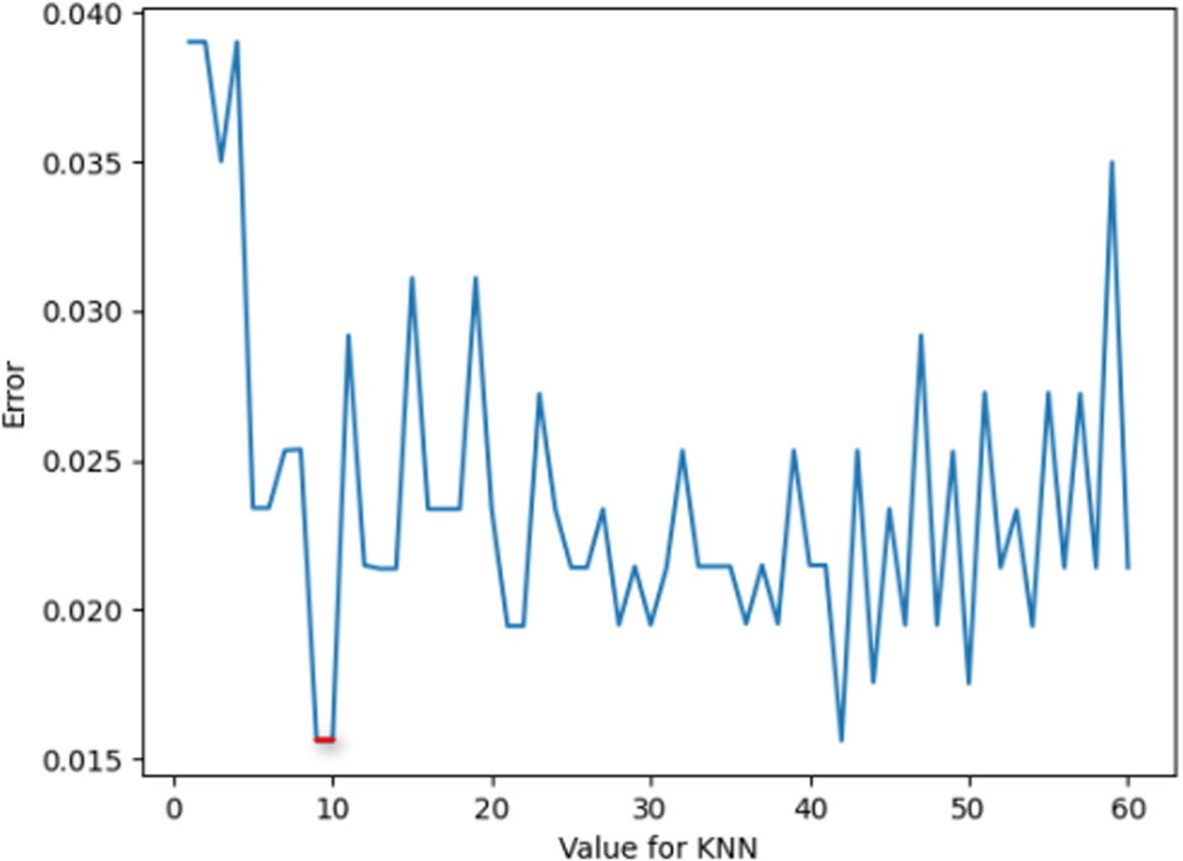
KNN parameter value error plot

As shown in Fig. 5, the marked part of the figure is the parameter value when the error is smallest; at this time, the value of K is 5, and the value of Weights is “uniform”.

In summary, the optimal parameters of KNN are taken as shown in Table 3.

**Table 3:** KNN parameter values

Parameter	Value
K	5
Weights	Uniform

#### DT parameter

In this experiment, the optimization of DT parameters includes the selection of the values of Max_Depth and Criterion.

**Fig. 6:**
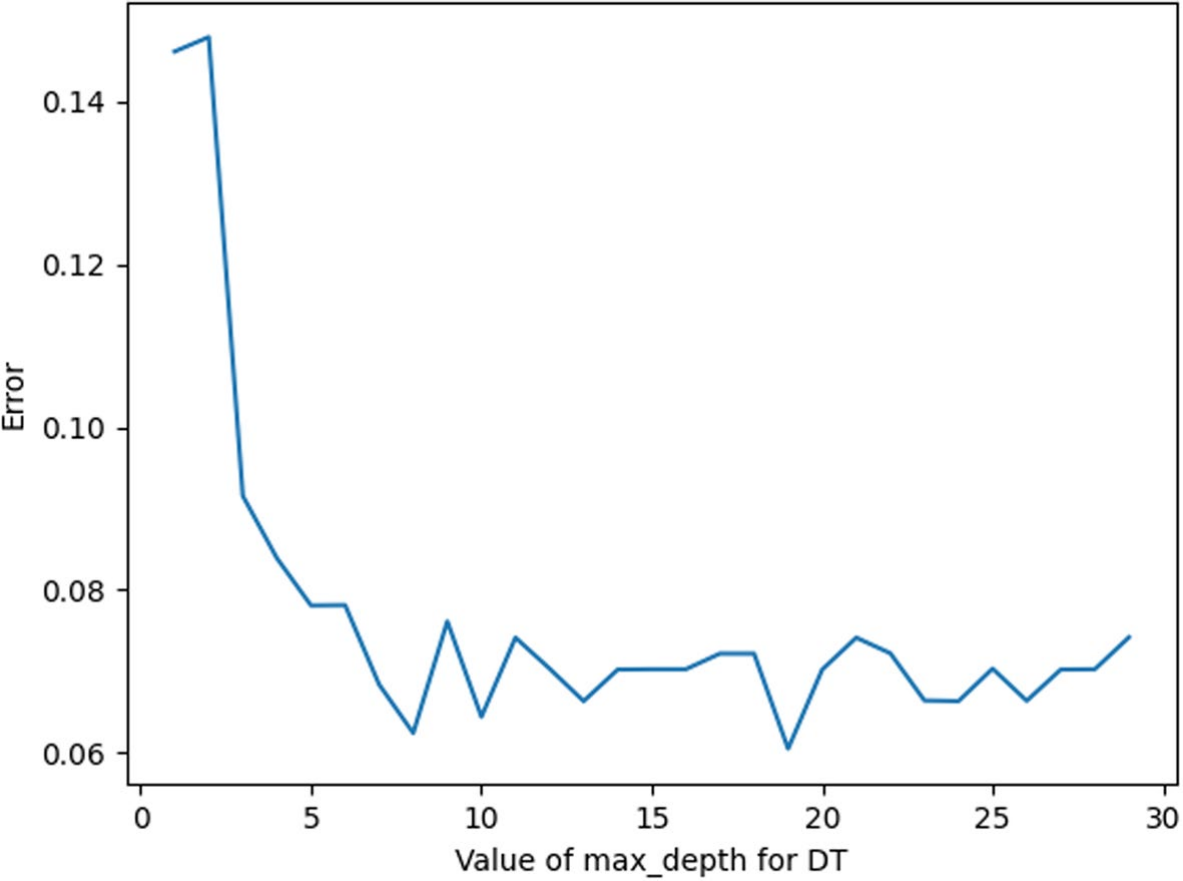
Max_Depth error plot

Max_Depth (depth of the constructed tree) parameter value selection is shown in Fig. 6. When the value of Max_Depth is 18, the corresponding error value is the smallest; thus, it is more appropriate to choose the value of Max_Depth as 18 in this experiment.

**Fig. 7:**
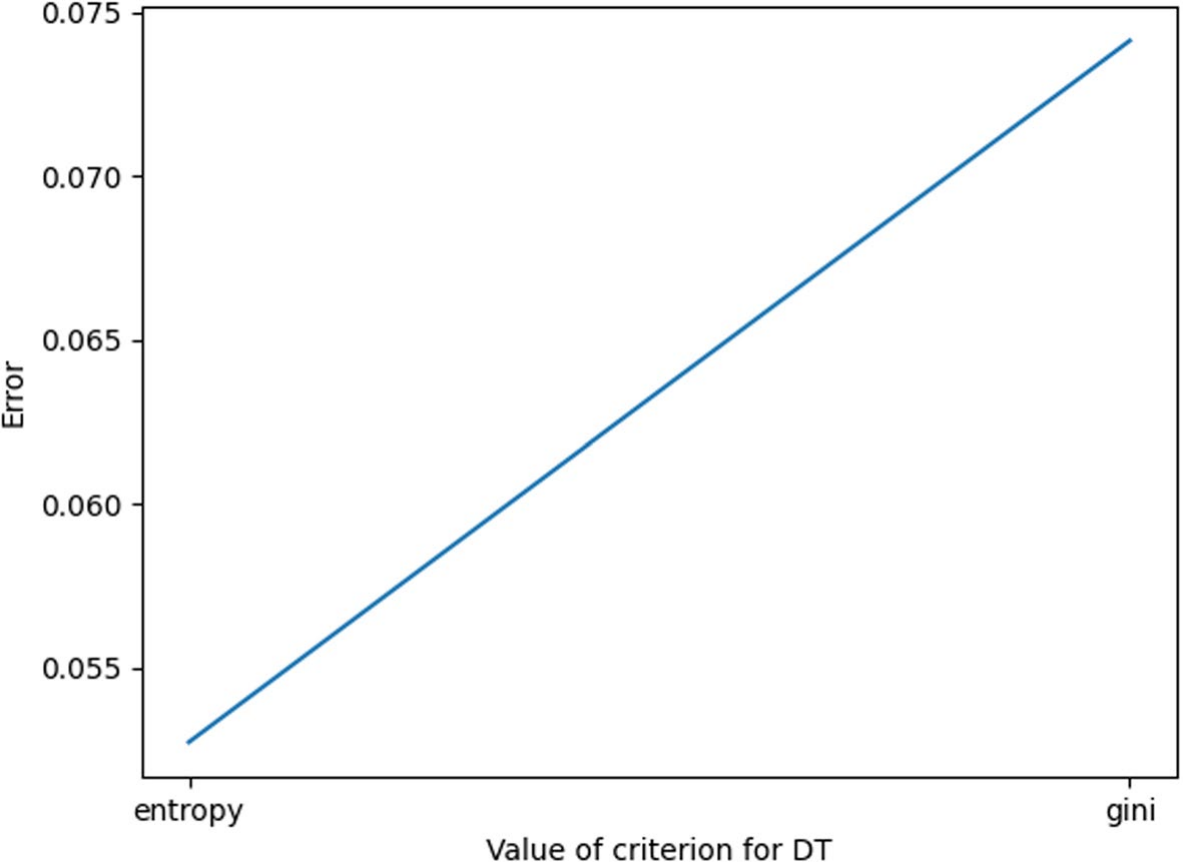
Criterion error plot

For Criterion, there are two general values of Criterion, namely,”entropy” and”gini”. The error is calculated according to the two different values, and the results are shown in Fig. 7. It can be seen that the error is smaller when the value of the Criterion parameter is”entropy”. Therefore, the value of”entropy” for Criterion in this experiment is more appropriate.

**Fig. 8:**
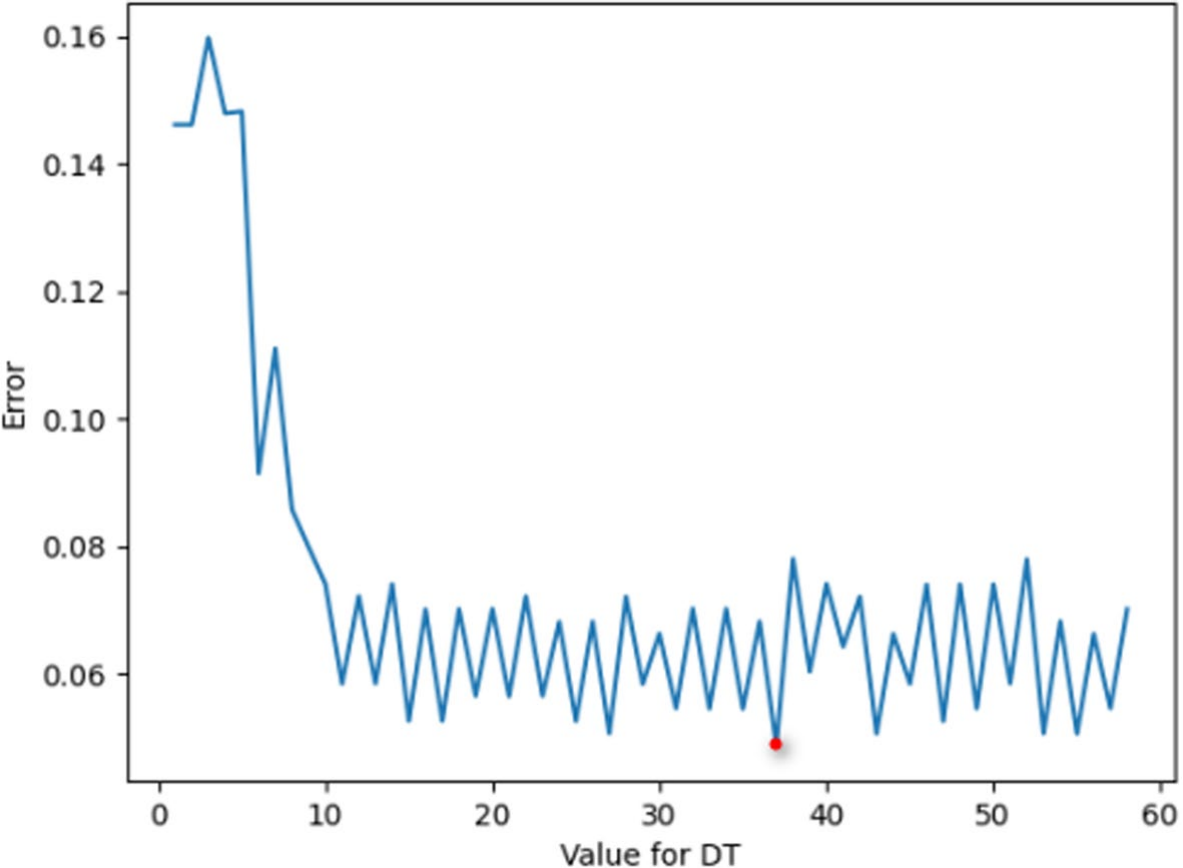
DT parameter value error plot

As shown in Fig. 8, the marked part of the figure is the parameter value when the error is smallest; at this time, the Max_Depth value is 18, and the Criterion value is “entropy”.

In summary, the optimal parameters of KNN are taken as shown in Table 4.

**Table 4:** DT parameter values

Parameter	Value
Max_Depth	18
Criterion	Entropy

#### SVM parameter

In this experiment, the optimization of the SVM parameters mainly includes the values of Kernel and C (penalty coefficient).

**Fig. 9:**
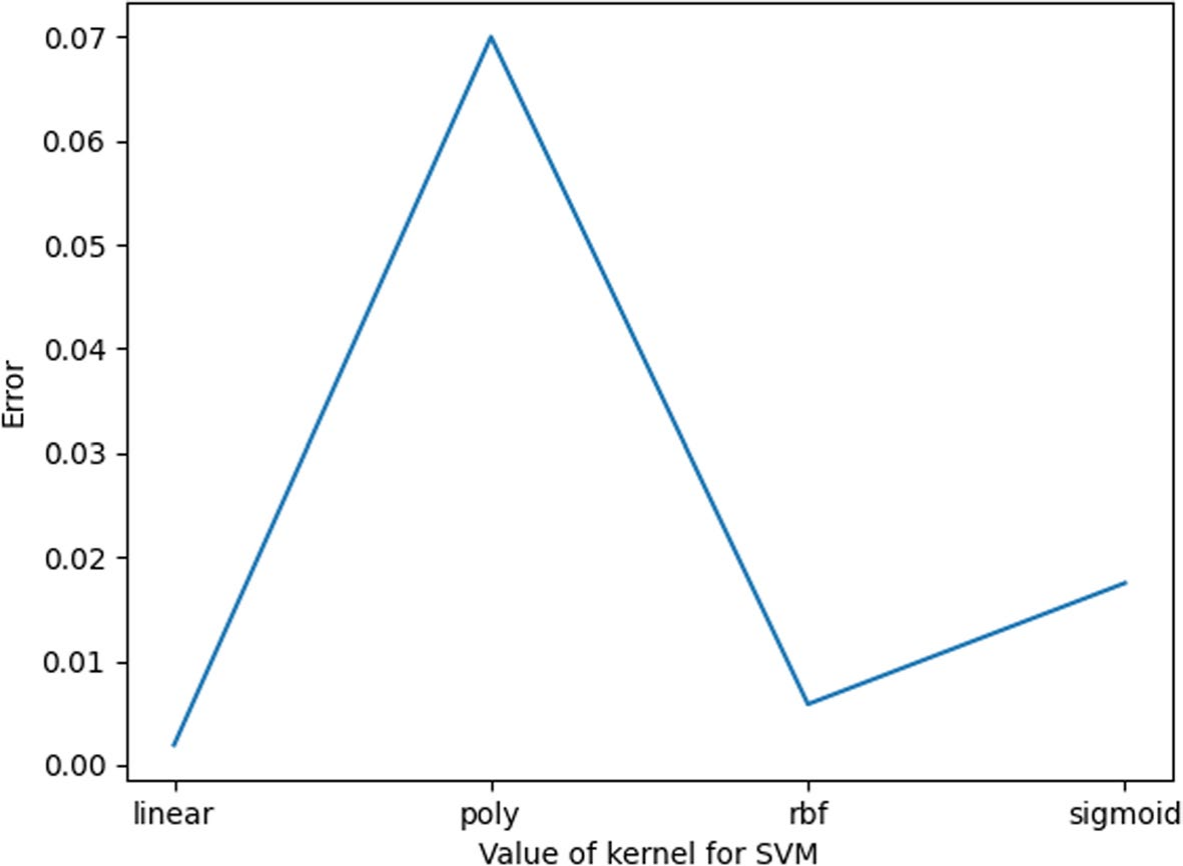
Kernel value error plot

For Kernel values, there are four general cases, namely, “linear”, “poly”, “rbf” and “sigmoid”. We calculate the error value for each of the four cases of the Kernel, as shown in Fig. 9. When the value of the Kernel is “linear”, the corresponding error value is the smallest; thus, it is more appropriate when the value of the Kernel is “linear” in this experiment.

**Fig. 10:**
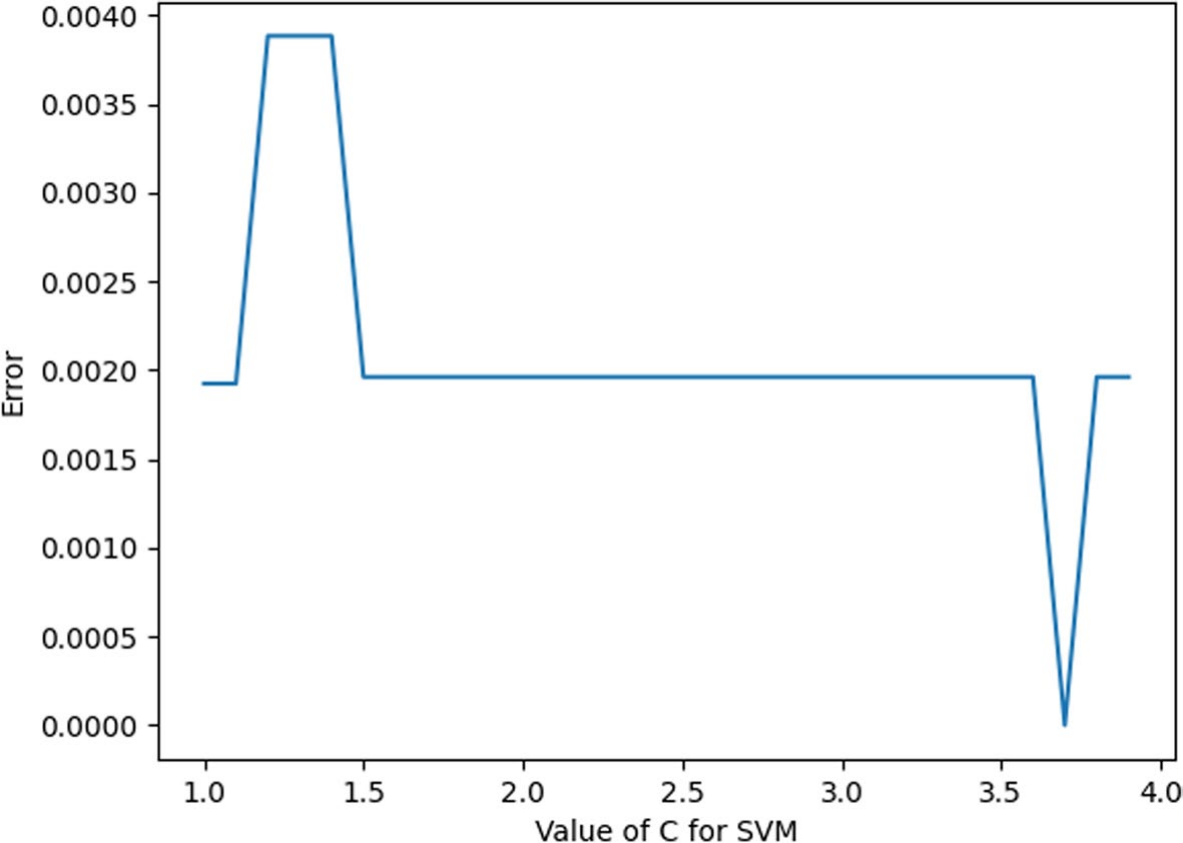
C value error plot

When the value of the Kernel is “linear”, the value of C (penalty coefficient) is discussed, and the error values corresponding to different values of C are shown in Fig. 10. When the value of C (penalty coefficient) is 3.7, the corresponding error value is the smallest; thus, the value of C (penalty coefficient) is 3.7 in this experiment, which is more appropriate.

In summary, the optimal parameters of KNN are taken as shown in Table 5.

**Table 5:** SVM parameter values

Parameter	Value
C	3.7
Kernel	Linear

## Results and discussion

### Evaluation indicator

In this study, we use six evaluation metrics to evaluate the model, including accuracy, recall, precision, F1 index, kappa coefficient and MCC coefficient. The details are shown below.

The confusion matrix is a matrix used to summarize the classification results of the classifier. It consists of true-positive TP, false-positive FP, false-negative FN and true-negative TN. The evaluation metrics of the model are calculated by TP, FP, FN and TN, which enables the performance evaluation of the model.

Accuracy, recall, precision, F1 index, Kappa coefficient and MCC coefficient. The calculation formula is shown in Eqs. 5–10.

5 $${\text{Accuracy}} = \frac{TP + TN}{{TP + TN + FP + FN}}*100\%$$

6 $${\text{Re}} call = \frac{TP}{{TP + FN}}*100\%$$

7 $${\text{Precision}} = \frac{TP}{{TP + FP}}*100\%$$

8 $${\text{F}}1 = \frac{{2*{\text{Re}} call*\Pr ecision}}{{{\text{Re}} call + \Pr ecision}}*100\%$$

9 $${\text{MCC}} = \frac{TP*TN - FP*FN}{{\sqrt {(TP + FP)(TP + FN)(TN + FP)(TN + FN)} }}{*}100{\text{\% }}$$

10 $$Kappa = \frac{{P_{0} - P_{e} }}{{1 - P_{e} }}*100\%$$

where $P_{0}$ represents the sum of the number of correctly classified samples in each category divided by the total number of samples. Suppose the number of real samples in each category is $x_{1} ,x_{2} ,x_{3} ,......,x_{n}$, the number of samples in each category predicted by the model is $y_{1} ,y_{2} ,y_{3} ,......,y_{n}$, and the total number of samples is n. The formula for $P_{e}$ is ${\text{P}}_{e} = \frac{{x_{1} *y_{1} + x_{2} *y_{2} + x_{3} *y_{3} + ...... + x_{n} *y_{n} }}{n*n}$^.^

In order to evaluate model performance in a balanced manner and to prevent specific data from influencing the results of performance evaluation, the following experiments were all conducted using 7–3 divided data (7–3) and tenfold cross−validation (10CV).

### Performance evaluation

#### Comparison between the same studies

In this section, the model is evaluated using the abovementioned metrics and compared with other models. The dataset uses the PIMA Indian diabetes dataset. The comparison results are shown in Tables 6 and 7. In the comparison, data for indicators not given by other researchers are replaced by "–". In Tables 6–12 and 14–20, the bold text indicates the experimental results of the method with the best performance under the current experimental evaluation metric.

**Table 6:** Model evaluation(7–3)

	Accuracy	Recall	F1 Index	Kappa	Precision	MCC
LR	0.774	0.782	0.761	0.527	0.626	0.538
KNN	0.638	0.567	0.549	0.149	0.576	0.174
SVM	0.754	0.723	0.730	0.466	0.760	0.478
NB	0.767	0.724	0.734	0.474	0.761	0.488
DT	0.877	0.868	0.866	0.733	0.821	0.733
RF	0.754	0.648	0.649	0.354	0.730	0.460
My model	**0.980**	**0.982**	**0.971**	**0.952**	**0.962**	**0.950**

**Table 7:** Model evaluation(10CV)

	Accuracy	Recall	F1 Index	Kappa	Precision	MCC
LR	0.719	0.674	0.719	0.357	0.615	0.362
KNN	0.655	0.573	0.655	0.163	0.546	0.184
SVM	0.692	0.640	0.692	0.290	0.579	0.297
NB	0.659	0.614	0.659	0.232	0.524	0.237
DT	0.896	0.884	0.896	0.768	0.871	0.774
RF	0.577	0.505	0.577	0.017	0.386	0.022
PSO-FCM [15]	0.954	0.956	–	–	0.955	0.908
PCA + K-Means + LR [12]	0.973	0.970	0.970	0.942	0.974	0.943
VAE + SAE With CNN [26]	0.923	–	–	–	–	–
K-Means + LR [11]	0.954	0.954	–	0.897	0.954	0.899
Conv-Lstm [24]	0.972	0.939	–	–	–	–
SVC [17]	0.790	0.700	0.715	–	0.731	–
LE [19]	0.750	0.720	0.730	–	0.730	–
X-BLR [29]	0.940	0.940	0.930	–	0.920	–
CGLSTM [30]	0.978	0.896	0.856	–	0.914	–
KFPredict [32]	0.935	0.980	–	–	0.850	–
My model	**0.981**	**0.984**	**0.980**	**0.962**	**0.977**	**0.965**

In Table 6, the proposed model has a 10–30% higher assessment metric than all other models. In Table 7, mean values were taken for comparison, with the proposed model having 0.3–23.1% higher accuracy, 0.4–28.4% higher recall, 1–26.5% higher F1 index, 2–6.5% higher kappa index, 0.3–24.7% higher precision, and 2.2–6.6% higher MCC index. Therefore, the proposed model in this paper outperforms the existing prediction models in all evaluation metrics and has better performance.

#### Comparison with other combinatorial classifiers

Since the combinatorial classifier stacking is used in this experiment, in this section, the model proposed in this thesis is compared with the latest published combinatorial classifier and the well-known bagging and boosting classifiers (the combinatorial classifier has been implemented using the Python language). If the researchers mention the values of their experimental parameters, the same parameter values are used. If no specific parameter values are indicated, all default values will be taken for the implementation. The specific experimental results are shown in Tables 8 and 9, and the results are analyzed and discussed separately.

**Table 8:** Model evaluation(7–3)

	Accuracy	Recall	F1 Index	Kappa	Precision	MCC
XGBoost [5]	0.877	0.849	0.864	0.732	0.977	0.754
REL [27]	0.974	0.975	0.973	0.946	0.952	0.946
GD [6]	0.896	0.886	0.889	0.778	0.875	0.778
ARS [7]	0.967	0.941	0.909	0.972	1.000	0.913
Bagging	0.922	0.904	0.916	0.833	0.980	0.841
Boosting	**0.982**	**0.990**	**0.984**	**0.969**	**0.958**	**0.969**
My model	0.980	0.982	0.971	0.952	0.962	0.950

**Table 9:** Model evaluation(l0CV)

	Accuracy	Recall	F1 Index	Kappa	Precision	MCC
XGBoost	0.883	0.843	0.859	0.724	0.958	0.747
REL	0.986	0.978	0.983	0.967	1.000	0.969
GD	0.924	0.917	0.915	0.830	0.893	0.833
ARS	**0.986**	**0.984**	**0.985**	**0.970**	**1.000**	**0.981**
Bagging	0.908	0.888	0.897	0.796	0.937	0.803
Boosting	0.982	0.976	0.982	0.961	0.967	0.962
My model	0.981	0.984	0.980	0.962	0.977	0.965

In Table 8, it can be seen that the boosting classifier, with the exception of the precision metric, outperforms the proposed model in all other metrics, 0.2% higher accuracy, 0.8% higher recall, 1.3% higher F1 index, 1.7% higher kappa index and 1.9% higher MCC index. Otherwise, the proposed models are better than the rest, with 0.6–10.3% higher accuracy, 0.7–13.3% higher recall, 5.5–10.7% higher F1 index, 0.6–22% higher kappa index, 1–8.7% higher precision, and MCC index 0.4–19.6% higher.

Table 9 shows that both REL and ARS classifier evaluation metrics are slightly better than the proposed model. These include 0.5% higher accuracy, 0.3–0.5% higher F1 index, 0.5–0.8% higher kappa index, 2.3% higher precision, and 0.1–1.6% higher MCC index. Otherwise, the proposed models are better than the rest, with 5.7–9.8% higher accuracy, 0.8–14.1% higher recall, 6.5–12.1% higher F1 index, 0.1–23.8% higher kappa index, and 1–8.4% higher precision, and the MCC index is 0.3–21.8% higher.

#### Comparison in the original dataset

In this section, the PIMA Indian diabetes dataset used is not preprocessed and includes noisy data such as missing values and extreme values. I will use the original PIMA Indian Diabetes dataset to evaluate the model proposed in this paper and to compare it with the models proposed by other researchers. The specific experimental results are shown in Tables 10 and 11.

**Table 10:** Model evaluation(7–3)

	Accuracy	Recall	F1 Index	Kappa	Precision	MCC
XGBoost	0.722	0.624	0.631	0.309	0.812	0.374
REL	0.645	0.507	0.403	0.007	0.500	0.028
GD	0.675	0.630	0.633	0.268	0.549	0.270
ARS	0.666	0.601	0.589	0.226	0.670	0.263
LR	0.571	0.534	0.533	0.067	0.383	0.067
KNN	0.683	0.645	0.647	0.296	0.552	0.296
SVM	0.735	0.700	0.697	0.395	0.579	0.396
NB	0.649	0.571	0.568	0.158	0.510	0.168
DT	0.632	0.592	0.592	0.184	0.462	0.184
RF	0.692	0.538	0.478	0.101	1.000	0.230
My model	**0.783**	**0.733**	**0.741**	**0.484**	**0.687**	**0.487**

**Table 11:** Model evaluation(10CV)

	Accuracy	Recall	F1 Index	Kappa	Precision	MCC
XGBoost	0.690	0.623	0.621	0.262	0.604	0.282
REL	0.551	0.530	0.520	0.052	0.381	0.055
GD	0.665	0.641	0.633	0.269	0.517	0.272
ARS	0.583	0.556	0.547	0.102	0.414	0.107
LR	0.567	0.547	0.536	0.082	0.397	0.087
KNN	0.718	0.673	0.673	0.352	0.613	0.359
SVM	0.654	0.634	0.626	0.257	0.504	0.260
NB	0.558	0.536	0.528	0.068	0.390	0.070
DT	0.673	0.647	0.640	0.283	0.528	0.286
RF	0.553	0.530	0.520	0.052	0.381	0.055
My model	**0.716**	**0.676**	**0.673**	**0.353**	**0.605**	**0.359**

In Table 10, the original PIMA Indian diabetes dataset is processed using the 7–3 division; the model proposed in this paper is superior to all other 11 models. The proposed model outperforms the rest of the models in terms of evaluation metrics, with 4.8–21.2% higher accuracy, 3.3–22.6% higher recall, 4.4–33.8% higher F1 index, 8.9–47.7% higher kappa index, and 1.7–30.4% higher precision, and 9.1–45.9% higher MCC index.

In Table 11, the performance of the KNN classifier is comparable to the performance of the proposed model in this paper, but the proposed model in this paper is more effective than the other models, where accuracy is 2.6–16.5% higher, recall is 0.3–14.6% higher, F1 index is 3.3–15.3% higher, kappa index is 7–30.1% higher, precision is 0.1–22.4% higher, and MCC index is 7.3–30.4% higher.

#### McNemar and standard deviation metrics

In this section, we will evaluate the model using McNemar and standard deviation. The McNemar test is based on a twofold continuous table of two model predictions, and the *P *value is the probability of observing this chi-squared value. The *P* value calculated by the test is lower than the given significance level, and the null hypothesis of equal performance of the two models can be rejected with a significant difference. Conversely, if the P-value calculated by the test is greater than the given significance level, the hypothesis of equal performance of the two models is supported, and there is no significant difference. The standard deviation, on the other hand, reflects the distribution among a set of data. We will analyze six models of LR, KNN, SVM, NB, DT, and RF with tenfold CV, given the significance value of 0.08, and the specific analysis results are shown in Tables 12 and 13.

**Table 12:** Standard deviation test

	Accuracy	Recall	F1 Index	Kappa	Precision	MCC
LR	0.05176	0.06968	0.07120	0.14293	0.36255	0.14600
KNN	0.07411	0.06519	0.07430	0.14713	0.19508	0.16640
SVM	0.05138	0.06302	0.06392	0.13108	0.15804	0.13834
NB	0.05985	0.06865	0.07093	0.13652	0.52460	0.13676
DT	0.03232	0.04472	0.03836	0.07525	0.09071	0.07240
RF	0.08375	0.08531	0.08912	0.17546	0.17179	0.18285
My model	**0.01365**	**0.01757**	**0.01652**	**0.03303**	**0.02258**	**0.03287**

**Table 13:** McNemar test

	1	2	3	4	5	6	7	8	9	10
LR	7.62e−06	6.10e−05	0.0220	0.0001	0.0009	0.0001	0.0002	0.0004	7.62e−06	0.0002
KNN	3.05e−05	2.38e−07	0.0001	5.72e−06	3.81e−06	1.09e−06	0.0002	6.10e−05	0.0009	7.62e−06
SVM	0.0001	3.81e−06	0.0070	4.00e−05	0.0001	0.0002	0.0002	1.90e−06	0.0001	6.10e−05
NB	4.76e−07	3.81e−06	0.0060	0.0009	1.52e−05	1.90e−06	0.0002	1.90e−06	7.62e−06	1.52e−05
DT	0.1250	0.0070	0.2180	0.1250	1.0000	0.0030	0.3750	0.0300	0.0600	0.0600

Regarding the PIMA Indian diabetes dataset, the standard deviation corresponding to each indicator of the model proposed in this paper is minimal, implying that the distribution among the data is relatively stable. At the same time, most of the data in Table 13 are smaller than the given significance threshold of 0.08, indicating a significant difference from the model presented in this thesis.

### Performance on other datasets

To further evaluate the performance of the present model and to demonstrate the reliability and applicability of the model, we tested it using a different dataset of diabetic patients, which was obtained from a direct questionnaire from patients of Sylhet Diabetes Hospital, Sylhet, Bangladesh.

#### Dataset description

The early diabetes risk prediction dataset was used in this experiment, which was obtained from a direct questionnaire from patients of Sylhet Diabetes Hospital, Sylhet, Bangladesh. Seventeen attributes were included in this dataset, 16 of which were related to diabetes diagnosis and 1 to a labeled attribute. The relevant attributes included age, gender, polydipsia, irritability, and weakness, and the labeled attributes were used to differentiate the diabetic population from the nondiabetic population. A total of 520 samples were included in the dataset, including 320 sample examples of diabetic patients and 200 sample examples of nondiabetic patients. The operations on the dataset are shown below.

#### Data collection prevention and experiment

Preprocessing of the dataset. Labeling of discrete data using 0/1 to facilitate later data processing. Quartile analysis was used to remove noise data such as extreme values and outliers. Feature selection was performed using Boruta’s algorithm to select the six most relevant features for diabetes prediction, namely, age, sex, polydipsia, irritability, sudden weight loss, and partial limb paralysis. The data were normalized using the Z-Score method. The preprocessed data were input to the K-Means++ unsupervised clustering algorithm for learning, and the clustering results were compared with the original data to count the number of correctly clustered data. Afterward, classification is performed using the ensemble learning stacking method, and the optimal parameters are found using a grid search.

#### Comparison between the same studies

In this section a comparison with traditional machine learning models is made. The comparison results are shown in Tables 14 and 15.

**Table 14:** Model evaluation(7–3)

	Accuracy	Recall	F1 Index	Kappa	Precision	MCC
LR	0.816	0.824	0.816	0.632	0.895	0.630
KNN	0.923	0.909	0.919	0.839	0.883	0.850
SVM	0.877	0.871	0.868	0.737	0.914	0.737
NB	0.946	0.932	0.942	0.885	0.918	0.891
DT	0.954	0.955	0.954	0.908	0.970	0.908
RF	0.900	0.891	0.900	0.796	0.855	0.808
My model	**0.986**	**0.986**	**0.985**	**0.970**	**0.988**	**0.971**

**Table 15:** Model evaluation(10CV)

	Accuracy	Recall	F1 Index	Kappa	Precision	MCC
LR	0.708	0.706	0.708	0.404	0.744	0.407
KNN	0.628	0.608	0.602	0.219	0.640	0.229
SVM	0.713	0.708	0.704	0.411	0.747	0.413
NB	0.853	0.836	0.842	0.689	0.809	0.708
DT	0.933	0.931	0.930	0.861	0.948	0.865
RF	0.589	0.580	0.589	0.157	0.630	0.161
My model	**0.945**	**0.947**	**0.945**	**0.887**	**0.972**	**0.892**

In Table 14, the proposed model outperformed the rest of the models in terms of evaluation metrics, with accuracy 3.2–17% higher, recall 3.1–16.2% higher, F1 index 3.1–16.9% higher, kappa index 6.2–33.8% higher, precision 1.8–13.3% higher, and MCC index 6.3–34% higher.

In Table 15, the proposed model outperforms the rest of the models in terms of evaluation metrics, including 1.2–35.6% higher accuracy, 1.6–36.7% higher recall, 1.5–35.6% higher F1 index, 2.6–19.8% higher kappa index, 2.4–34.2% higher precision, and 2.7–18.4% higher MCC index.

#### Comparison between combined classifiers

As above, the models in this thesis are compared with different combinatorial classifiers. The comparison results are shown in Tables 16 and 17.

**Table 16:** Model evaluation(7–3)

	Accuracy	Recall	F1 Index	Kappa	Precision	MCC
XGBoost	0.954	0.952	0.953	0.907	0.944	0.908
REL	0.969	0.971	0.968	0.937	0.986	0.938
GD	0.931	0.948	0.925	0.852	1.000	0.861
ARS	0.786	0.774	0.778	0.559	0.771	0.566
Bagging	0.961	0.961	0.959	0.919	0.975	0.919
Boosting	0.984	0.983	0.984	0.969	0.972	0.969
My model	**0.986**	**0.986**	**0.985**	**0.970**	**0.988**	**0.971**

**Table 17:** Model evaluation(10CV)

	Accuracy	Recall	F1 Index	Kappa	Precision	MCC
XGBoost	**0.977**	**0.974**	**0.975**	**0.951**	**0.963**	**0.953**
REL	0.662	0.657	0.653	0.309	0.696	0.311
GD	0.922	0.920	0.918	0.837	0.940	0.840
ARS	0.497	0.492	0.489	-0.010	0.557	-0.010
Bagging	0.915	0.918	0.911	0.825	0.981	0.835
Boosting	0.935	0.933	0.932	0.866	0.945	0.869
My Model	0.945	0.947	0.945	0.887	0.972	0.892

In Table 16, the proposed model outperforms the rest of the models in terms of evaluation metrics, including 0.2–20% higher accuracy, 0.3–21.2% higher recall, 0.1–20.7% higher F1 index, 0.1–41.1% higher kappa index, 0.2–21.7% higher precision, and MCC index 0.2–40.5% higher.

As seen from Table 17, the XGBoost classifier is slightly better than the model in this thesis in all aspects, including 3.2% higher accuracy, 2.7% higher recall, 3% higher F1 index, 6.4% higher kappa index, 0.9% higher precision and 6.1% higher MCC index. In addition, the model in this thesis outperforms the other combinatorial classifiers, which includes 1–28.3% higher accuracy, 1.4–29% higher recall, 1.3–29.2% higher F1 index, 2.1–57.8% higher kappa index, 2.7–27.6% higher precision and 2.3–58.1% higher MCC index.

#### Comparison on the original dataset

As above, we also compared the performance of the different models on the original early diabetes risk prediction dataset. The comparison results are shown in Tables 18 and 19. In the comparison, data for indicators not given by other researchers are replaced by "–".

**Table 18:** Model evaluation(7–3)

	Accuracy	Recall	F1 Index	Kappa	Precision	MCC
XGBoost	0.846	0.837	0.839	0.678	0.863	0.679
REL	0.846	0.830	0.837	0.678	0.807	0.694
GD	0.801	0.796	0.788	0.576	0.872	0.579
ARS	0.564	0.516	0.499	0.034	0.600	0.038
LR	0.602	0.534	0.533	0.073	0.675	0.074
KNN	0.532	0.521	0.517	0.042	0.647	0.042
SVM	0.634	0.589	0.590	0.184	0.694	0.186
NB	0.724	0.647	0.641	0.336	0.696	0.421
DT	0.794	0.783	0.783	0.566	0.833	0.566
RF	0.673	0.567	0.525	0.161	0.662	0.254
My model	**0.858**	**0.863**	**0.854**	**0.710**	**0.919**	**0.714**

**Table 19:** Model evaluation(10CV)

	Accuracy	Recall	F1 Index	Kappa	Precision	MCC
XGBoost	0.794	0.696	0.642	0.335	0.726	0.386
REL	0.615	0.650	0.503	–	0.615	0.0
GD	0.801	0.700	0.654	0.346	0.747	0.386
ARS	0.615	0.650	0.503	–	0.613	0.0
LR	0.615	0.650	0.503	–	0.615	0.0
KNN	0.586	0.510	0.437	0.057	0.631	0.081
SVM	0.615	0.650	0.503	–	0.615	0.0
NB	0.615	0.650	0.503	–	0.615	0.0
DT	0.809	0.707	0.665	0.369	0.763	0.405
RF	0.615	0.650	0.503	–	0.615	–
My model	**0.825**	**0.721**	**0.683**	**0.403**	**0.782**	**0.432**

Table 18 shows that the proposed model achieves better results than the rest of the models. In particular, the accuracy is 1.2–32.6% higher, the recall is 2.6–34.7% higher, the F1 index is 1.5–35.5% higher, and the kappa index is 3.2–37.4% higher, the accuracy is 4.7–31.9% higher, and the index is 2–29.3% higher MCC.

Table 19 shows that the proposed model outperforms the rest of the models in terms of all assessment indicators. The accuracy rate is 1.6–23.9% higher, the recall rate is 1.4–21.1% higher, the F1 index is 1.8–24.6% higher, the kappa index is 3.4–34.6% higher, the precision is 1.9–16.9%, and the MCC index is 2.7–35.1% higher.

#### McNemar and standard deviation metrics

As above, the model was also evaluated using McNemar and standard deviation. The comparison results are shown in Tables 20 and 21.

**Table 20:** Standard deviation test

	Accuracy	Recall	F1 Index	Kappa	Precision	MCC
LR	0.06284	0.06673	0.06321	0.12658	0.08191	0.12877
KNN	0.07060	0.06287	0.06292	0.12460	0.10125	0.13311
SVM	0.05091	0.05672	0.05214	0.10452	0.07093	0.10687
NB	0.05719	0.05612	0.05876	0.11560	0.06858	0.11265
DT	**0.03185**	**0.03485**	**0.03385**	**0.06773**	**0.04322**	**0.06832**
RF	0.07624	0.07751	0.07754	0.15251	0.09912	0.15300
My Model	0.04418	0.05473	0.04875	0.09707	0.06765	0.09527

**Table 21:** McNemar test

	1	2	3	4	5	6	7	8	9	10
LR	0.0002	0.0100	0.0650	0.1090	0.0120	0.0100	0.0004	0.0009	0.0210	0.0010
KNN	7.62e−05	6.10e−05	0.0070	0.0110	0.0070	0.1430	3.81e−06	1.90e−06	0.0100	0.0009
SVM	0.0004	0.0002	0.0380	0.0700	0.0200	0.0650	0.5400	0.0200	0.0004	0.0009
NB	0.1250	0.015	0.1090	0.0310	0.0100	0.0300	0.1090	0.0300	0.0004	0.0700
DT	0.2500	0.5000	0.0300	0.1250	0.0150	0.0300	0.0380	0.6870	0.0070	0.0002
RF	4.005e−05	1.90e−06	0.0030	0.0009	2.38e−07	1.19e−07	7.62e−06	1.52e−05	1.90e−06	6.10e−05

On the early diabetes risk prediction dataset, DT has the smallest standard deviation, implying that the distribution among the data are relatively stable. However, it does not imply a better performance than the model in this thesis. Most of the data in Table 21 are smaller than the given significance threshold of 0.08, indicating a significant difference from the model presented in this thesis.

### Computational complexity analysis

In this section, I will analyze the feature selection algorithm, clustering algorithm, data processing and computational complexity of stacking used in the paper. The computational complexity is divided into two main parts: time complexity (time occupied by CPU) and space complexity (memory occupied space), and the results of the analysis are as follows.

#### Data processing

Data processing mainly includes the processing of noisy data such as missing values, extreme values, and outliers. This part uses the WEKA data analysis tool, which first cleans the data and later normalizes the cleaned data so that the data fall within a specific range of values.

#### Feature selection

The computational complexity of feature selection is shown in Table 22 below.

**Table 22:** Computational complexity analysis of feature selection

	Time complexity (s)	Space complexity (MIB)
PIMA Dataset	6.250	4.30
Early Diabetes Risk Prediction Dataset	7.901	4.40

#### Clustering

The computational complexity of clustering is shown in Table 23 below.

**Table 23:** Computational complexity analysis of clustering

	Time complexity (s)	Space complexity (MIB)
PIMA Dataset	0.258	4.30
Early Diabetes Risk Prediction Dataset	0.169	4.30

#### Stacking

The computational complexity of stacking is shown in Tables 24 and 25.

**Table 24:** Computational complexity analysis of stacking (7–3)

	Train (s)	Test (s)	Total Time (s)	Total space (MIB)
PIMA Dataset	0.153	0.014	0.168	4.30
Early Diabetes Risk Prediction Dataset	1.049	0.137	1.187	4.30

**Table 25:** Computational complexity analysis of stacking (10 CV)

	Train (s)	Test (s)	Total Time (s)	Total space (MIB)
PIMA Dataset	25.063	0.423	25.486	4.30
Early Diabetes Risk Prediction Dataset	15.685	0.465	16.150	4.30

### Analysis of model advantages and disadvantages

#### Advantages

Compared with other models, the reasons for the better performance of the diabetes prediction model based on Boruta feature selection and ensemble learning proposed in this paper are as follows:

(1) In this paper, a suitable feature selection algorithm is used. In the field of diabetes prediction, we believe that the use of feature selection algorithms is not only to reduce the feature dimensionality, but more importantly to select features that are useful for diabetes diagnosis. If characteristics are selected simply in pursuit of better index results, but they will not be used in the physician's diagnosis of diabetes, then we consider them to be meaningless and not applicable to the actual diagnosis. If the features selected are those used in the diagnosis of diabetes, even if the proposed method predicts poor index results, we can improve the results by tuning the parameters or changing to a different algorithm. This is because these are the characteristics that can really help doctors make a diagnosis of diabetes. Therefore, the Boruta feature selection algorithm is finally selected by conducting comparative experiments in this paper.

(2) The unsupervised clustering algorithm is used. The unsupervised clustering algorithm can divide the data into different sets of data clusters. Using correctly segmented data can improve the accuracy, precision and other metrics of diabetes data classification, as well as reduce the model training time.

(3) This model uses ensemble learning and tunes the parameters of the model. In other research works, they mostly use single classifier models. However, the results of single classifier prediction are highly susceptible to the influence of data. In addition, most of the researchers did not adjust the parameters of their models.

Based on the above three points, this method was compared with other methods in experiments on two datasets. The experimental results show that although the results of certain experimental metrics are lower than other methods, the difference is minimal. And most of the experimental metrics are superior to other methods. This is fully illustrated by the detailed data comparison in the experimental part of this paper as well.

#### Disadvantages

(1) From the description of the two datasets, it can be seen that both datasets used in this paper have the problem of imbalance of data sample points. However, we do not address the data imbalance problem in the data preprocessing stage in this paper, which may result in a "majority class" preference in the trained model.

(2) The amount of data used for model training in this paper is insufficient. The PIMA diabetes dataset has 768 data and the early diabetes risk prediction dataset has 520 data. This may lead to inadequate training of the model. Therefore, future work will focus on finding a large and realistic diabetes dataset.

In this section, we focus on evaluating the performance of the model. First, it was evaluated on the commonly used diabetes dataset, PIMA, including the use of accuracy, precision, recall, F1 index, kappa coefficient and other metrics evaluated, and good performance was achieved. Second, using the early diabetes risk prediction dataset, the same good performance was achieved, verifying the reliability and applicability of the model. Finally, the computational complexity of the feature selection, clustering, and stacking methods and the advantages and disadvantages of the models are analyzed on two datasets.

In summary, the model has good performance, indicating that the results of this study are promising. Its research results are mainly applied to clinical screening and early warning of early diabetes. In the future, it may be involved in the preliminary diagnosis of other diseases and will be widely used in the field of bioinformatics.

## Summary and future work

The difficulty of artificial intelligence technology in predicting whether a person is a diabetic population is how to improve the accuracy of the prediction results. In this paper, we propose a new method for predicting diabetes based on Boruta feature selection and ensemble learning, which mainly consists of extracting relevant features of the dataset using the Boruta feature selection algorithm, discovering some potential K patterns in the data using the K-Means++ algorithm, supervising the classification of the data using stacking, and optimizing the parameters using grid search to find the optimal values of the parameters. We used the PIMA Indian diabetes dataset for our experiments and achieved 98% accuracy using tenfold cross validation. In addition, comparing this model with other models, this model performs better. To validate the performance of the model on other datasets, we evaluated the model using the early diabetes risk prediction dataset, all with good results. Therefore, the model has strong applicability and reliability. It is successful in the early prediction of diabetic disease.

Although the model works better, there are two aspects of the two datasets used in this experiment. On the one hand, the sample size of the dataset is small, and the attribute values and noise in the data are lower than in the real data. On the other hand, there is an imbalance in the sample size ratio between diabetic and nondiabetic populations in the dataset. When the model is trained, it will be biased to the category with a high sample size, so that the category with a low sample size is not adequately trained, resulting in lower model performance. For future work, first, it is necessary to cooperate with hospitals and use the hospital data as training data, while the ratio of diabetic population and nondiabetic population data samples should be kept equal. Second, the number of datasets will be expanded to ensure sufficient training and testing. We will also work on other chronic diseases, such as heart disease and kidney disease.

## Data Availability

The experimental part conducted in this paper contains a total of 2 datasets, the PIMA diabetes dataset and the early diabetes risk prediction dataset. Both diabetes datasets are from the UCI Data Knowledge Base and were publicly available diabetes datasets at the time of the experiments conducted in this thesis. The data that support the findings of this study are available from [Baidu.com], but restrictions apply to the availability of these data, which were used under license for the current study and thus are not publicly available. However, the data are available from the corresponding author upon reasonable request and with permission of [Baidu.com]. The datasets generated and/or analyzed during this study are available in the [UC Irvine Machine Learning] repository [https://archive.ics.uci.edu/ml/machine-learning-databases/00529/]. The experiments and the datasets used in this paper are within the scope of the law.
